# C_60_- and CdS-Co-Modified Nano-Titanium Dioxide for Highly Efficient Photocatalysis and Hydrogen Production

**DOI:** 10.3390/ma17051206

**Published:** 2024-03-05

**Authors:** Meifang Zhang, Xiangfei Liang, Yang Gao, Yi Liu

**Affiliations:** 1 Institute of Carbon Neutral New Energy Research, Yuzhang Normal University, Nanchang 330031, China; mfzhang@whu.edu.cn (M.Z.); ygao2023@126.com (Y.G.); 2 School of Chemical and Environmental Engineering, Wuhan Polytechnic University, Wuhan 430023, China

**Keywords:** co-modified, CdS-C_60_/TiO_2_, photocatalytic activity, hydrogen evolution

## Abstract

The inherent properties of TiO_2_, including a wide band gap and restricted spectral response range, hinder its commercial application and its ability to harness only 2–3% of solar energy. To address these challenges and unlock TiO_2_’s full potential in photocatalysis, C_60_- and CdS-co-modified nano-titanium dioxide has been adopted in this work to reduce the band gap, extend the absorption wavelength, and control photogenerated carrier recombination, thereby enhancing TiO_2_’s light-energy-harnessing capabilities and hydrogen evolution capacity. Using the sol-gel method, we successfully synthesized CdS-C_60_/TiO_2_ composite nanomaterials, harnessing the unique strengths of CdS and C_60_. The results showed a remarkable average yield of 34.025 μmol/h for TiO_2_ co-modified with CdS and C_60_, representing a substantial 17-fold increase compared to pure CdS. Simultaneously, the average hydrogen generation of C_60_-modified CdS surged to 5.648 μmol/h, a notable two-fold improvement over pure CdS. This work opens up a new avenue for the substantial improvement of both the photocatalytic degradation efficiency and hydrogen evolution capacity, offering promise of a brighter future in photocatalysis research.

## 1. Introduction

In recent years, photocatalytic technology has garnered significant attention from researchers owing to its cost-effectiveness, simplicity, and minimal environmental impact. This versatile technology leverages a range of photocatalysts to disintegrate a wide array of both organic and inorganic pollutants, offering the promise of converting CO_2_ into valuable renewable chemicals and facilitating water decomposition [1,2,3]. Among these photocatalysts, titanium dioxide (TiO_2_) stands out for its relatively high activity, rendering it highly effective in the purification of air and water, even in biomedical applications [4,5]. Consequently, its applications in the field of photocatalysis hold considerable promise [6,7].

Nevertheless, the inherent limitations of TiO_2_ are its wide band gap and restricted spectral response range, which result in its ability to utilize only approximately 2–3% of solar energy and hinder its commercial application [8]. To address these challenges and unlock the full potential of TiO_2_ in photocatalysis, modification techniques have emerged as a pivotal approach to enhancing its photocatalytic performance. These techniques aim to reduce the band gap, extend the absorption wavelength, and control photogenerated carrier recombination, thereby enhancing TiO_2_’s light-energy-harnessing capabilities [9]. Notably, the choice of dopants significantly influences the photocatalytic activity, affecting the recombination of photogenerated electrons (e^−^) and holes (h^+^) on the material’s surface.

In the field of photoelectrochemical water decomposition, nano-TiO_2_ coupling systems have been extensively researched [10,11]. Cadmium sulfide (CdS), with its narrow energy gap of 2.4 eV, has proved effective as a coupling agent for TiO_2_, promoting the photocatalytic decomposition of water [12,13]. The strong coupling between TiO_2_ and CdS allows for the direct combination of TiO_2_ particles with dispersed CdS particles. Although CdS exhibits photoetching instability when used independently, the formation of composite nanomaterials with TiO_2_, characterized by a wider band gap, effectively reduces the likelihood of photogenerated electron–hole recombination. Thus, the strategic coupling of TiO_2_ with CdS mitigates the photoetching instability associated with CdS alone and expands the spectral response range [14,15,16].

Fullerene (C_60_), renowned for its exceptional electron (e^−^) transport capabilities, has emerged as an intriguing option for mitigating charge recombination. This has fueled increasing interest in TiO_2_ catalysts supported by C_60_ [17,18]. C_60_ boasts an extensive three-dimensional π electron system, featuring a closed core–shell structure comprising 60 delocalized π electrons and 30 bonded molecular orbitals. Even minor structural variations or changes in the solvent properties can influence the electron transfer within the system, making the incorporation of C_60_ an attractive strategy for enhancing effective electron transfer [19,20]. With a band gap ranging from 1.6 to 1.9 eV, C_60_ holds considerable promise for practical applications, and catalysts modified with C_60_ are poised to achieve a remarkable photocatalytic performance.

Recognizing the complementary advantages of CdS and C_60_, we have adopted a comprehensive approach to co-modifying TiO_2_. Utilizing the sol-gel method, we have successfully synthesized CdS-C_60_/TiO_2_ composite nanomaterials with highly effective photocatalytic properties. Remarkably, the hydrogen production of CdS modified with C_60_ exhibited a two-fold increase compared to pure CdS, reaching an average of 5.648 μmol/h. Furthermore, TiO_2_ co-modified with CdS and C_60_ demonstrated an impressive average yield of 34.025 μmol/h, representing a staggering 17-fold increase over pure CdS. This study underscores the synergistic benefits of combining CdS and C_60_ in enhancing the photocatalytic and hydrogen production performance of TiO_2_, opening up new avenues for sustainable and efficient environmental remediation processes.

## 2. Materials and Methods

**Materials**: The cadmium chloride (CdCl_2_, 99.0%) and sodium sulfide (Na_2_S·9H_2_O) were purchased from Tianjin Kemiou Chemical Reagent Co., Ltd. (Tianjin, China). The Malachite Green (MG), P25 (Degussa, Hamburg, Germany), and butyl titanate (Ti(OBu)_4_) were all purchased from China National Pharmaceutical Group Chemical Reagent Co., Ltd. (Beijing, China). The C_60_ was purchased from Shanghai Macklin Biochemical Co., Ltd. (Shanghai, China). All the other reagents were analytical-grade. The pH of the solution was adjusted using nitric acid and sodium hydroxide solutions. All experiments used secondary water.

**Synthesis of CdS**: Dissolve 3.1148 g of CdCl_2_·2.5H_2_O in 20 mL of ethanol in the first bottle, and dissolve 2.4004 g of Na_2_S·9H_2_O in 20 mL of ethanol in the second bottle. After stirring for 10 min, the second bottle is added to the first bottle slowly, drop by drop. Gel A is stirred for two hours at room temperature before being transferred into an alumina crucible. Place the sample into a muffle furnace and set the conditions to increase from room temperature to 450 °C, with a heating rate of 2 °C/min in an air atmosphere, and maintain it there for 2 h after aging at room temperature for 24 h. The calcination conditions are the same for each sample below. Finally, create a sample of CdS powder by grinding the acquired sample in an agate mortar [21,22,23].

**Synthesis of CdS-TiO_2_**: Make gel A as directed by the conditions listed above. In a flask with a circular bottom and labeled “gel B”, combine 20 mL of butyl titanate (TBT) with 30 mL of ethanol solution. The combination added dropwise is stirred at room temperature for 2 h. Prepare another combination using 20 mL of ethanol, 4 mL of distilled water, 2 mL of nitric acid, and 20 mL of gel A. They should be added dropwise to the room-temperature gel B solution, stirred as they are added, and left to react for 24 h. Age for 24 h in an alumina crucible. The sample is burned under the same conditions in a muffle furnace and then ground to produce a CdS-TiO_2_ sample.

**Synthesis of CdS-C_60_**: In order to create CdS, 30 mg of Ox-C_60_ (also known as activated C_60_) is added to a combination of distilled water and agitated for 2 h. An alumina crucible is used to age gel C for 24 h. Then, under the same conditions, it is calcined in a muffle furnace. The CdS-C_60_ sample is obtained by grinding it after cooling to room temperature.

**Synthesis of CdS-C_60_-TiO_2_**: Synthesize gel B and gel C separately, measure 20 mL of ethanol and 4 mL of distilled water, and combine 2 mL of nitric acid and gel C to put together another mixture. The combination dropwise is added to the gel B solution at room temperature, stir whilst adding, and react for 24 h. Age it in an alumina crucible for 24 h and burn it in a muffle furnace underneath the identical conditions. Grind to achieve the CdS-C_60_-TiO_2_ sample. The experimental characterization is detailed in the Supporting Information (pages 2–4).

## 3. Results and Discussion

**Figure 1:**
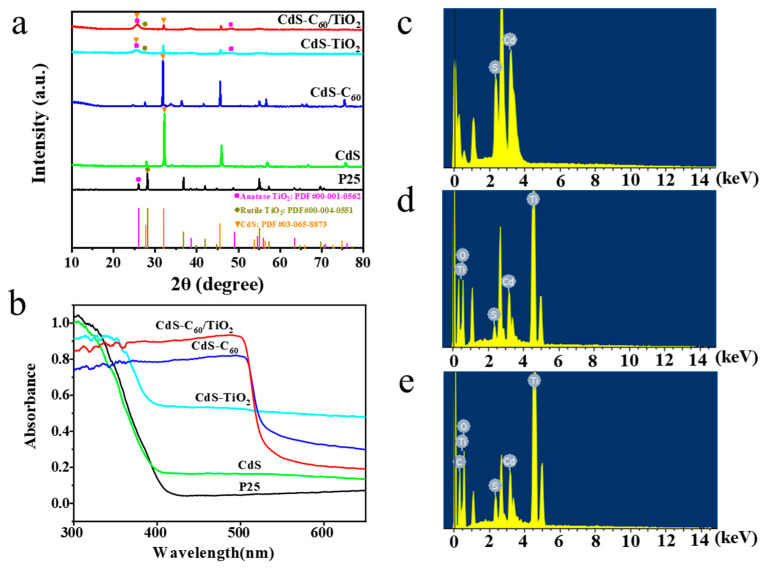
(**a**) XRD patterns and (**b**) UV–Vis diffuse reflectance spectra of P25, CdS, CdS-C_60_, CdS-TiO_2_, and CdS-C_60_/TiO_2_. Energy-Dispersive X-ray (EDX) elemental microanalysis of (**c**) CdS, (**d**) CdS-TiO_2_, (**e**) CdS-C_60_/TiO_2_.

To explore the changes in the TiO_2_ crystal morphology after the modification, we conducted X-ray diffraction (XRD) measurements for the pure TiO_2_ and the modified TiO_2_ materials in Figure 1a. As can be seen, the diffraction peaks of P25 (commercial TiO_2_) appear at 2*θ* = 25.08°, 37.94°, 48.14°, 54.26°, 55.26°, 62.90°, 68.98°, 70.02°, and 75.24°, respectively, corresponding to the diffraction peaks of anatase-phase TiO_2_ (101), (004), (200), (105), (211), (204), (116), (200), and (215), respectively, where 2*θ* = 25.08° was the strongest diffraction peak. The diffraction peaks of the rutile phase (110), (101), and (200) crystal faces appear at 2*θ* = 27.7°, 36.2°, and 42.82°, indicating that TiO_2_ is a mixed crystal structure dominated by anatase phases and rutile phases [24]. Comparing P25, CdS-TiO_2_, and CdS-C_60_/TiO_2_, the rutile diffraction peak weakened in turn, and the anatase-phase diffraction peak increased in turn, indicating that the introduction of CdS into the samples can inhibit the growth of their rutile phase. As we all know, rutile TiO_2_, as the most stable crystalline structure form, has a good crystallization state and fewer defects, resulting in electrons and holes that are easy to compound, with almost no photocatalytic activity. Therefore, the CdS-C_60_/TiO_2_ composite with less rutile TiO_2_ will have a better catalytic performance. We also tested the XPS patterns of P25, CdS-TiO_2_, and CdS-C_60_/TiO_2_, as shown in Figure S1 (Supporting Information). It was proven that there is no Ti2p separation between the C_60_, CdS, and TiO_2_ phases after preparation, but the shift in the oxygen atom toward a high binding energy means that the chemical bonds between the oxygen atom and other elements become tighter and more stable, which indicates that CdS-C_60_ has a significant effect on the modification of TiO_2_, which promotes the transfer of electrons between oxygen atoms and other elements, thereby increasing the activity of the catalyst.

Not only that but most UV radiation with a wavelength of less than 400 nm is absorbed by P25. The absorption bands of CdS, CdS-C_60_, CdS-TiO_2_, and CdS-C_60_/TiO_2_ all entered the visible light region. This is mainly because the band gap of CdS is 2.4 eV, the response characteristics of visible light are better, and the crystal structure of the original substance is changed after being modified with CdS. Compared with pure CdS and pure TiO_2_, the absorbance of CdS-C_60_ and CdS-C_60_/TiO_2_ increased, showing that the band gaps of various semiconductor catalysts overlapped in the ultraviolet and visible regions, respectively, which is conducive to improving their photocatalytic performance. The C_60_-modified sample’s absorption band edge shows some redshift, indicating that C_60_ can narrow the catalyst’s band gap, which is due to the interaction between the components, causing surface lattice oxygen vacancy and other defects [25,26,27]. The results above indicate that the crystal structure of TiO_2_ is alterable by CdS and C_60_, leading to the creation of defects on the surface lattice. The cause of the edge absorption is the defect absorption rather than the actual absorption of the lattice [28]. Additionally, the band gap energy of TiO_2_ is decreased, enabling it to be activated by visible light and to exhibit photocatalytic activity. These changes not only enhance the utilization of light energy but also improve the overall efficiency of the photocatalysis process. Meanwhile, EDX fluorescence spectroscopy analyses on each sample were performed to detect the type and content of the characteristic elements contained in each sample, as shown in Figure 1c–e.

**Figure 2:**
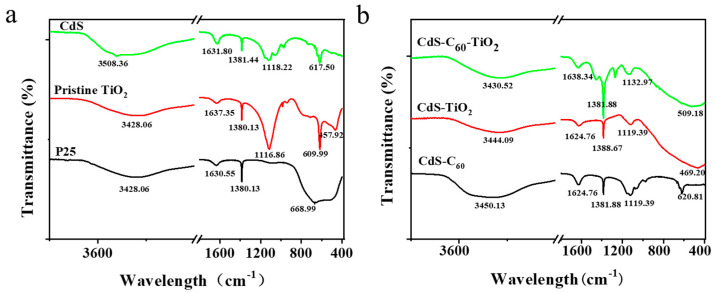
(**a**) FT–IR spectra of P25, pristine TiO_2_, CdS, (**b**) CdS-C_60_, CdS-TiO_2_, and CdS-C_60_-TiO_2_.

As the FT-IR spectra show in Figure 2a,b, all the catalyst samples have strong absorption peaks at 3420–3510 cm^−1^ and 1620–1640 cm^−1^, corresponding to the bending vibration peaks of the H-O-H bonds, which indicates the bending vibration of the presence of numerous water molecules adsorbed onto the catalyst surface. These peaks confirm that water molecules adhere to the catalyst surface in a physical manner. Consequently, it can be concluded that these nanomaterials possess the ability to exhibit a significant photocatalytic effect, which may be the presence of the majority of hydroxyl groups on the surface, and under photoexcitation, the e^−^-h^+^ pair generated by the catalyst sample acts on the hydroxyl group to generate highly oxidizing ·OH. The abundant and diverse hydroxyl groups present on the surface also promote the creation of sites where O_2_, CO_2_, and CO molecules can be adsorbed [29]. The absorption peak at 1380 cm^−1^ of the spectrum indicates that O_2_ molecules are adsorbed on the surface. Whether it is the formation of highly oxidizing free radicals or the provision of adsorption sites for small molecules, this facilitates the oxidation of organic material into a variety of small molecule compounds via photocatalysis. In comparison to P25, the absorption peaks of CdS-TiO_2_ and CdS-C_60_/TiO_2_ shift toward higher frequencies. The peak at 450–510 cm^−1^ signifies the presence of titanate TiO_3_^2−^, indicating that the transition of TiO_2_ involves lattice distortion [30]. It can be deduced that the periodic potential field distortion generated by the modification of CdS and C_60_ caused lattice defects in the TiO_2_. The TiO_2_ band gap decreases, leading to an enhanced ability to catalyze visible light. Although the sample contains a small amount of titanate TiO_3_^2−^, the photocatalytic activity can be effectively improved by introducing more crystal defects [31]. The band of CdS-C_60_-TiO_2_ changes the lattice to produce some characteristic peaks at 1380 cm^−1^, which further indicates that CdS, C_60_, and TiO_2_ are bonded, changing the original lattice structure of the TiO_2_. It may be inferred from band analyses of CdS, CdS-C_60_, CdS-TiO_2_, and CdS-C_60_/TiO_2_ that the same absorption peak, which may represent a Cd-S bond vibration, is present in all of these materials.

Since the photocatalytic activity of the functionalized materials is largely dependent on their morphological structure, it can be found that P25 (Figure S2a,b, Supporting Information) is a solid microsphere. However, after being modified by CdS, the particles become less uniform, and as seen using XRD, there is an interaction between CdS and TiO_2_, making its crystal lattice larger. CdS and C_60_ uniformly cover the surface of TiO_2_ in the form of lumps and microspheres, and the aggregation of small particles aggravates the surface roughness (Figure S2c–j, Supporting Information), as is consistent with the results of SEM. By comparing the TEM of the samples CdS-TiO_2_ (Figure S3a, Supporting Information) and CdS-C_60_/TiO_2_ (Figure S3b, Supporting Information), we see the crystal plane of the CdS-C_60_/TiO_2_ composite is clearer and regular, the crystal form is more perfect, and the lattice fringes of TiO_2_ and CdS can be seen clearly. This indicates that the introduction of C_60_ changed the lattice structure of TiO_2_, making the crystal form better. In addition, the connection between the suitable specific surface area and the variables affects the sample’s photocatalytic performance. We calculated the pore size and pore distribution of each sample using the Barrett–Joyner–Halenda technique and the Halsey equation, as shown in Table S1 (Supporting Information) [32,33,34]. From the nitrogen adsorption data of the six catalyst samples, when compared to CdS, the specific surface area of CdS-TiO_2_ has increased in comparison to commercial P25 and CdS-C_60_, but the rate of the change in the pore size is much greater than the change in the specific surface area. This may be a result of the dopant entering the lattice and causing the pore size to decrease, conducive to producing lattice defects in the crystal structure. This improves the photocatalytic activity [35]. As a product of CdS and C_60_ co-modification, in CdS-C_60_/TiO_2_, it is hypothesized that the factors influencing the photocatalytic performance of the sample are related to an appropriate specific surface area and a certain degree of lattice defects. As the specific surface area increases, so does the number of active sites, leading to the higher adsorption performance of the catalyst. Conversely, reducing the specific surface area leads to a decrease in both the pore volume and pore size. The presence of certain lattice defects proves beneficial in enhancing the light-harnessing effect of TiO_2_ [36].

**Figure 3:**
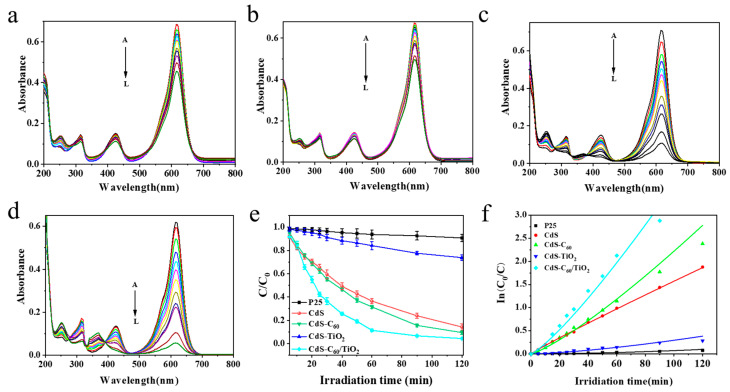
Spectrum of adsorption of MG solution in the presence of (**a**) CdS; (**b**) CdS-TiO_2_; (**c**) CdS-C_60_; and (**d**) CdS-C_60_/TiO_2_ under different irradiation times when exposed to halogen tungsten lamp. For A–G, each absorbance spectrum was recorded over a 5 min interval; for G–J, each absorbance spectrum was recorded over a 10 min interval; for J–L, each absorbance spectrum was recorded over a 30 min interval with visible light illumination. (**e**) Absorbance variations as a function of irradiation time (**f**), ln (c_0_/c), and the linear of control for P25, CdS, CdS-C_60_, CdS-TiO_2_, CdS-C_60_/TiO_2_ in MG deterioration after 120 min exposure to radiation at ambient temperature. [MG] = 4 mg/L; [P25] (CdS, CdS-C_60_, CdS-TiO_2_, CdS-C_60_/TiO_2_) = 0.6 g/L.

By assessing the rate at which MG degrades under 120 min of visible light irradiation, the CdS- and C_60_-co-modified TiO_2_ samples can be evaluated for their visible light catalytic activity. They were put through photocatalytic degradation tests with commercial P25 under identical circumstances to assess their photocatalytic activity. The UV absorption spectra of CdS, CdS-C_60_, CdS-TiO_2_, and CdS-C_60_/TiO_2_ are displayed in Figure 3a–d. These spectra demonstrate that the absorbance values decrease with an increasing visible light irradiation period, indicating a significant and quick degradation of MG. To examine the kinetics of MG’s photocatalytic breakdown in more detail, we measured the photocatalytic degradation curves of five catalyst samples, as shown in Figure 3e–f [37]. According to the relevant formula, we calculated the apparent rate constant *k*_app_ for the catalytic reactions with different catalysts in Table S1 (Supporting information), and it was indicated that the photocatalytic efficiency of all the samples was significantly higher than that of P25 when exposed to visible light. The order of photocatalytic efficiency was CdS-C_60_/TiO_2_ > CdS-C_60_ > CdS > CdS-TiO_2_ > P25. It can be seen that the composite materials have significant catalytic activity. Compared with the pure materials, the catalytic activity of the C_60_-modified materials is improved due to C_60_’s ability to enhance the quantum efficiency and facilitate charge transfer: C_60_ can enhance the efficiency of separating photogenerated e^−^-h^+^ pairs. Meanwhile, it can also enhance the adsorption efficiency during the degradation process [38,39]. This is also due to the small band gap width (2.4 eV) of CdS, which can induce photocatalysis in the visible light region. CdS can provide excited e^−^ to TiO_2_, while water and oxygen are used to generate hydroxyl radicals (·OH) and superoxide anion radicals (·O_2_^−^). These free radicals contribute to the photooxidation of the adsorbed organic matrix. The CdS-C_60_/TiO_2_ sample exhibits both the characteristics of C_60_ and CdS, thus exhibiting the highest photocatalytic activity.

Moreover, we conducted adsorption mechanical analysis on the different catalysts. The pore size and pore distribution can be calculated in Table S2 (Supporting Information). The factors influencing the photocatalytic performance of the sample are connected to the combined impact of an appropriate specific surface area and a certain level of lattice defects, which is beneficial for improving the optical effect of TiO_2_. The results of our adsorption-based linear fitting analysis are consistent with the desorption equilibrium data for MG using different catalysts, as shown in Figure S4 (Supporting Information). One can enhance the TiO_2_ by increasing its specific surface area and modifying its crystal structure; TiO_2_ co-modified with C_60_ can be expected to improve in its photocatalytic performance; and given sufficient time, CdS-C_60_ is comparable to CdS-C_60_-TiO_2_ in terms of its catalytic performance.

**Figure 4:**
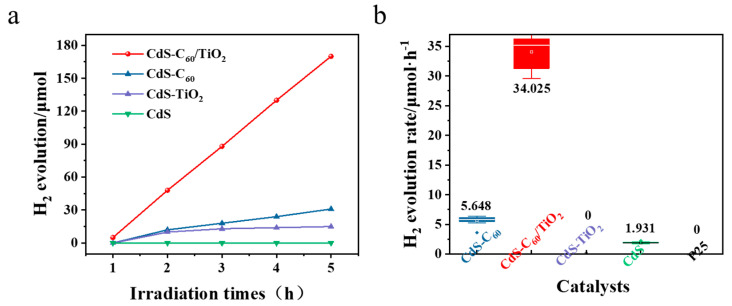
(**a**) Several kinds of hydrogen production with a time change map; (**b**) different catalysts per hour of hydrogen production.

To further analyze the catalytic performance of each catalyst, we conducted a comparison of the photocatalytic hydrogen production yield over time using 10% lactic acid as a sacrificial reagent. This comparison included various catalysts under visible light conditions, as illustrated in Figure 4a. We tested the average hydrogen production of different catalysts per hour under continuous illumination for 5 h in Figure 4b. As illustrated in Figure 4a,b, the hydrogen production performance of these photocatalysts in order from best to worst is CdS-C_60_/TiO_2_ > CdS-C_60_ > CdS > CdS-TiO_2_ = P25. The visible light hydrogen production effect of pure P25 and CdS-modified TiO_2_ is basically zero, and the average hydrogen production of C_60_-modified CdS is 5.648 μmol/h. The photocatalytic activity of CdS when co-modified with TiO_2_ and C_60_ is approximately twice as high as that of pure CdS, whereas the average yield of TiO_2_ co-modified with CdS and C_60_ is 34.025 μmol/h, which is roughly 17 times higher than that of pure CdS. The narrow band gap of C_60_ enables the photocatalyst to have a significantly broader absorption band range, thereby enhancing its light absorption capability in the visible light spectrum. C_60_ has good conductivity and can detach photogenerated e^−^ and promote good separation of the photogenerated e^−^-h^+^, so the visible light hydrogen production performance in CdS can be enhanced through modification with C_60_. Furthermore, both CdS and C_60_ demonstrate significant light absorption capabilities within the visible spectrum, and C_60_ as an electronic relay can promote the flow of CdS and TiO_2_ photogenerated e^−^. C_60_ can be used as an active site for photocatalytic hydrogen production, specifically the catalytic cleavage of water to produce H_2_. This indicates that TiO_2_ co-modified with CdS and C_60_ has a high photocatalytic activity under visible light conditions.

A typical impedance equivalent plot is shown in Figure S4a (Supporting Information) to demonstrate the impact of the charge transfer on the activity of catalysts. The EIS Nyquist plots of different catalysts are shown in Figure S5b (Supporting Information). In the Nyquist plots of EIS, the semicircle’s diameter shows that CdS, CdS-C_60_, CdS-TiO_2_, and CdS-C_60_/TiO_2_ have a lower charge transfer resistance compared to pure TiO_2_. The order of decreasing resistance values is R(A) < R(B) < R(C) < R(D) < R(E) < R(F). It can speed up the charge separation and promote e^−^ transfer. This helps to decrease the likelihood of recombining e^−^-h^+^ pairs and enhances the photocatalytic activity [40,41,42]. From the results of electrochemical AC impedance, we see the effective charge transfer rate of CdS is weaker than that of CdS-TiO_2_, but in the photodegradation experiment, the photodegradation rate is higher than that of CdS-TiO_2_. The possible reasons for this are that CdS can provide excitation of e^−^ to TiO_2_; within 18 h after the modification of the empty gold sheet, the pure CdS is unstable on its own, and it may have developed photocorrosion, which, in turn, reduced the conductivity.

**Figure 5:**
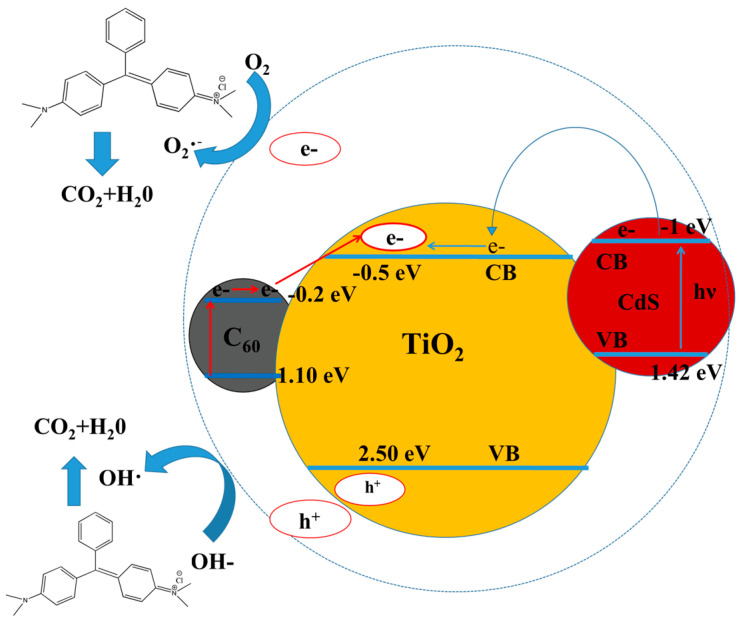
The photodegradation mechanism schematic for CdS-C_60_-TiO_2_.

To illustrate the photodegradation mechanism of the catalyst CdS-C_60_-TiO_2_, we give a schematic diagram of the corresponding photodegradation mechanism, as shown in Figure 5. On the one hand, C_60_ has a good electron transport performance, which can accelerate the photoinduced charge separation and slow down charge recombination. Under natural light irradiation, the valence electrons (e^−^) in TiO_2_ are excited to the conduction band (CB), and holes (h^+^) are generated in the valence band (VB). However, these photogenerated electrons–holes can recombine quickly in the photocatalytic reaction, but since C_60_ is a good electron acceptor and can receive electrons from the titanium dioxide, the lifetime of the photogenerated e^−^ and h^+^ will be extended during the transfer process. The photogenerated h^+^ can form hydroxyl radicals, with a strong oxidizing activity and OH^−^ adsorbed on the surface of the TiO_2_. The photogenerated e^−^ form oxyanion radicals with the oxygen molecules adsorbed on the surface of the TiO_2_, which can effectively photolyze organic radicals or water under the action of free radicals. The resulting active substances (•O^2−^ and •OH) have a high degradation activity for MG.

On the other hand, TiO_2_ and CdS can transition under illumination simultaneously, with the latter’s conduction band position being higher than the former, so there is a misalignment of the two in the schematic diagram of the mechanism. Generally, when TiO_2_ absorbs light with an energy greater than its band gap, the e^−^ in the valence band can be excited and move to the conduction band. This leads to the formation of highly active e^−^ in the conduction band and corresponding h^+^ in the valence band [43]. The photogenerated e^−^ and h^+^ are separated and moved toward the surface of the semiconductor particles due to the presence of an electric field, causing highly active photogenerated e^−^-h^+^ pairs. This indicates that the transition of the electrons from the excited state of CdS to the conduction band of TiO_2_ occurs, and then these e^−^ are absorbed by O_2_ molecules to form the superoxide anion radical O_2_^−^ [44,45,46]. Consequently, the adsorbed organic matrix’s photooxidation characteristics are improved by this shift in vector transfer, to effectively enhance the photooxidation performance of the adsorbed organic matrix. Due to the photogenerated h^+^’s valence potential, which prevents them from oxidizing hydroxyl groups into hydroxyl radicals, CdS photocorrodes to create Cd^2+^. In short, both C_60_ and CdS components enhance the catalytic effect of TiO_2_; therefore, CdS-C_60_/TiO_2_ exhibits a high catalytic activity.

## 4. Conclusions

In summary, the preparation of CdS- and C_60_-co-modified TiO_2_ photocatalysts via the sol-gel method was followed by their comprehensive characterization using various techniques. Our rigorous investigation into their photocatalytic performance and hydrogen production capacity showcased the exceptional effectiveness of the ternary composite catalyst CdS-C_60_/TiO_2_ in oxidizing the targeted degradants. The results demonstrated the impressive average hydrogen production of 34.025 μmol/h for TiO_2_ co-modified with CdS and C_60_, marking a substantial 17-fold increase when compared to pure CdS. In parallel, the average hydrogen generation for C_60_-modified CdS surged to 5.648 μmol/h, representing a notable two-fold enhancement over pure CdS. This remarkable performance can be attributed to the collaborative impact of CdS and C_60_, which displayed synergistic effects when combined with TiO_2_. The co-modifying strategy effectively minimized the likelihood of photogenerated electron–hole recombination while simultaneously reducing the material’s band gap. These findings underscore the vast potential of CdS-C_60_/TiO_2_ as a high-performance photocatalytic system, with versatile application fields such as renewable energy and environmental remediation. We have compiled a comprehensive overview of highly efficient catalysts for the degradation of MG (refer to Table S3 in the Supporting Information). Although our designed catalysts may not exhibit an optimal performance, our work offers a practical approach to enhancing the effectiveness of photocatalytic degradation by utilizing novel ternary materials.

## Data Availability

Data are contained within the article and supplementary materials.

## Supplementary Materials

The following supporting information can be downloaded at https://www.mdpi.com/article/10.3390/ma17051206/s1. Figures S1–S5: XPS, SEM, TEM, N_2_ adsorption and desorption isotherms and pore size distributions and electrochemical impedance spectroscopy measurement of different samples; Tables S1–S3: Kinetics parameters of photocatalytic degradation of MG, physical parameters of the adsorption and the photocatalytic degradation of MG at different catalysts. References [47,48,49,50,51,52,53,54,55] are cited in the supplementary materials.
