# Supplementary material for: C60- and CdS-Co-Modified Nano-Titanium Dioxide for Highly Efficient Photocatalysis and Hydrogen Production
Source: Materials (Basel). 2024 Mar 5;17(5):1206. doi: 10.3390/ma17051206 (PMC10934443; doi:10.3390/ma17051206)
Supplement: Supplementary file 1 [file materials-17-01206-s001.zip › materials-2805728-supplementary.pdf]

Supporting Information

C60- and CdS-Co-Modified Nano-Titanium Dioxide for Highly Efficient Photocatalysis and Hydrogen Production

Meifang Zhang [1], Xiangfei Liang [1,*], Yang Gao [1] and Yi Liu [2]

1 Institute of Carbon Neutral New Energy Research, Yuzhang Normal University, Nanchang 330031, China;

mfzhang@whu.edu.cn (M.Z.); ygao2023@126.com (Y.G.)

2 School of Chemical and Environmental Engineering, Wuhan Polytechnic University, Wuhan 430023, China

* Correspondence: xfliang96@126.com (X.L.); yiliuchem@whu.edu.cn (Y.L.)

EXPERIMENTAL CHARACTERIZATION

XRD characterization was performed on a D8X advanced X-ray diffractometer (Bruker Company, Germany) using the powder method and the X-ray source being Cu Kα， λ= 1.5406 Å, tube voltage of 40 KV, tube current of 40 mA, step width of 0.02 degrees. XPS characterization was performed on a Nexsa X-ray photoelectron spectrometer (Thermofisher Company, USA) using Al K Alpha as source gun type, 400 um as focused X-ray beam size, 0.05 eV as energy step size and 401 as numbers of energy steps. The characterization of UV visible diffuse reflectance spectroscopy is carried out on a UV 2550 (SHIMADZU Corporation Kyoto, Japan) spectrometer, using $BaSO_4$ as a reference and Kubelka Munk equation to convert reflectance intensity into absorption intensity, thereby determining the bandgap width of the sample. The infrared spectrum was tested using a 360 fourier transform infrared spectrometer (FT-IR, Thermo Nicolet) to analyze the changes in surface groups and chemical bonds in the crystal structure of doped titanium dioxide nanomaterials.

Microscopic morphology analysis was performed using scanning electron microscopy (FE-SEM, SEM-DEX, JSM-5610LV, Japan Electron Society) and transmission electron microscopy (JEOL, JSM-2010). Apply SEM to observe the surface morphology of sample particles, determine the average size of particles, and determine the distribution of sample particles in the matrix; At the same time, energy dispersive X-ray fluorescence spectroscopy EDX was measured to analyze the elemental composition on the surface of the sample; TEM can directly observe the surface morphology and crystallization of the sample material, determine the average size and particle size distribution of particles, and study the interface atomic structure of the sample. BET characterization is performed by degassing the sample under vacuum at 120 °C for 3 hours, and then collecting data on a surface and porous analyzer of the microscopics ASAP 2020 model at a liquid nitrogen temperature of 77 K in a nitrogen atmosphere. Calculate and analyze the specific surface area and pore size distribution of the sample through BET and BJH.

Photocatalytic MG degradation: In a specially designed double-layer U-shaped

reaction vessel (Pyrex heat-resistant glass reactor), 0.15 g of catalyst sample and 250 mL of MG solution are added. The vessel compartment is filled with circulating water to maintain a constant temperature of 25 °C, and a magnetic stirrer is installed at the bottom of the vessel. Use a 450 W halogen tungsten lamp with a 460 nm filter as the light source, which shields light with a wavelength less than 460 nm, ensuring that the reaction system is all visible light. The photodegradation experiment takes MG as the target degradation material, and under dark conditions, stir for 5 min to achieve adsorption desorption equilibrium; During the operation, the first 13 samples were collected every 5 min from the sampling holes of the reactor to collect 4 mL of suspension. The interval between the last two samples was 0.5 h, and after centrifugation (8000 rpm, 4 min), their absorbance was measured on a UV spectrometer. The absorbance was converted into solution concentration to obtain the degradation rate.

Photocatalytic degradation began after turning on the visible light lamp. The concentration of the MG solution was analyzed at specific times (in all 5 h) to determine the photocatalytic degradation efficiency for each composite. The typical reference TiO_2 was used for comparison. The following equation was used to compute the deterioration efficiency:

$$\varphi = \left(\frac{c - c_0}{c}\right) \times 100\% \quad (1)$$

where is the MG solution's rate of degradation, c_0 is the dye's initial concentration, and c is the dye concentration after degradation. The photocatalytic degradation is supposed to follow pseudo-first-order reaction kinetics in the studied concentration range. Reaction kinetics has often been described in terms of Langmuir-Hinshelwood model, which can be expressed as [3]:

$$r = -\frac{\mathrm{d}c}{\mathrm{d}t} = k_r \frac{K_a c}{1 + K_a c} \quad (2)$$

where r is the oxidation rate, c in the concentration of the reactant, t is the

illumination time, (-dc/dt) is the degradation rate of MG, k_r is a reaction rate constant; K_a is the adsorption coefficient of the reactant. At low initial dye concentration (c_0 is small), the rate was proportional to the dye concentration and the rate constant was observed to be a pseudo-first-order constant [4]. Accordingly, Eq. 2 can be changed to Eq. 3.

$$\ln \frac{c_0}{c} = k_r K t = k_{app} t \quad (3)$$

where k_{app} is the apparent rate constant, used as the basic kinetic parameter for different photocatalysts, since it makes it possible to calculate the concentration of residual MG in the solution as well as the photocatalytic activity independent of the preceding adsorption period in the dark.

Hydrogen production performance test: To analyze the catalytic performance of each catalyst, we conducted a comparison of the photocatalytic hydrogen production yield over time using 10% lactic acid as a sacrificial reagent. Lactic acid is a common sacrificial agent in the system of photocatalytic hydrogen production. The sacrificial agent acts as an electron donor. In this process, the oxidation potential of lactic acid is higher than that of H_2O, increasing the driving force of the oxidation reaction and reducing the recombination of photo generated electrons. It can consume photo generated holes and leave electrons to react with water to produce hydrogen.

Separately weigh a positive quantity of catalyst pattern and add it to a difficult glass tube reactor containing 100 mL of Na_2S (0.25M)-Na_2SO_3 (0.35 M) mixture. Conduct ultrasonic remedy for 15 minutes, seal with nitrogen fuel and vacuum. Use a 300 W xenon lamp as the mild supply (CHF-XM-250 W, Beijing Trusttech Co.), and filter out ultraviolet mild the usage of a Kenko L-42 filters. To further measure the quantity of hydrogen generated the use of a gasoline chromatograph (GC, SP-6800A, TCD, five Å molecular sieve column, argon service gas).

Impedance experiment: The tests were performed on a CHI660C electrochemical system (Shanghai Chenhua, China) using a standard three-electrode cell with a

working electrode (gold film), a platinum wire was used as the counter electrode, and the Ag/AgCl was used as reference electrode. The electrochemical impedance spectroscopies (EIS) were carried out in an electrolyte solution (pH=7.4) in the presence of 5 mM Fe $[CN]_6^{3-/4-}$ and 0.1 M KCl with the open circuit potential of 0.24V. The detailed experiment steps can be seen as followed. Firstly, in order to prepare the gold film, we have used the fisher BK7 (catalog no. 12-540-A) glass slides as the glass substrates. Then several tiny drops of CdS-C_{60}-TiO_2, CdS-C_{60}, CdS-TiO_2, CdS and P25 solution (500 mg/mL) were dropped to cover the surface of the bare gold film electrodes separately. After several hours, the electrodes were washed by ultrapure water several times and dried in nitrogen flow. At last, the immobilization of catalyst tiny layer was fabricated and the electrodes can be used in the experiments followed.

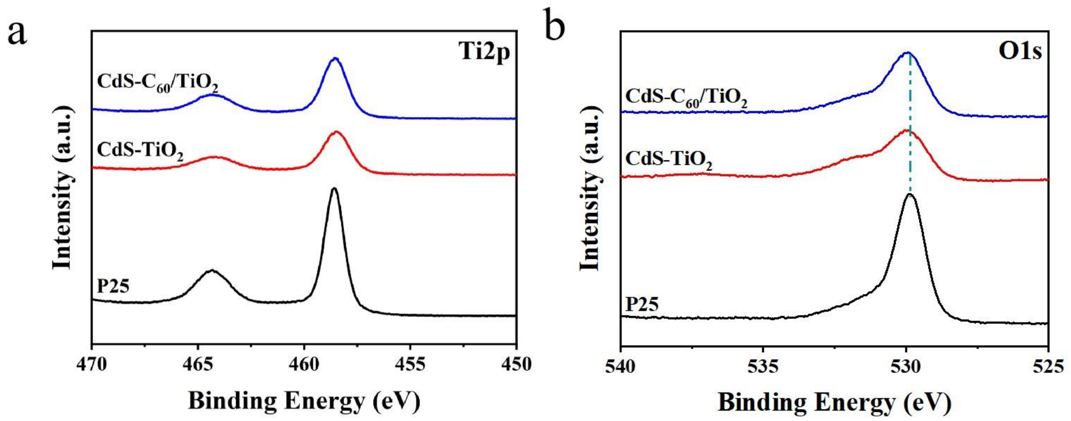

Figure S1. High-resolution XPS spectrums of Ti2p (a) and O1s (b) for P25, CdS-TiO_2 and CdS-C_{60}/TiO_2.

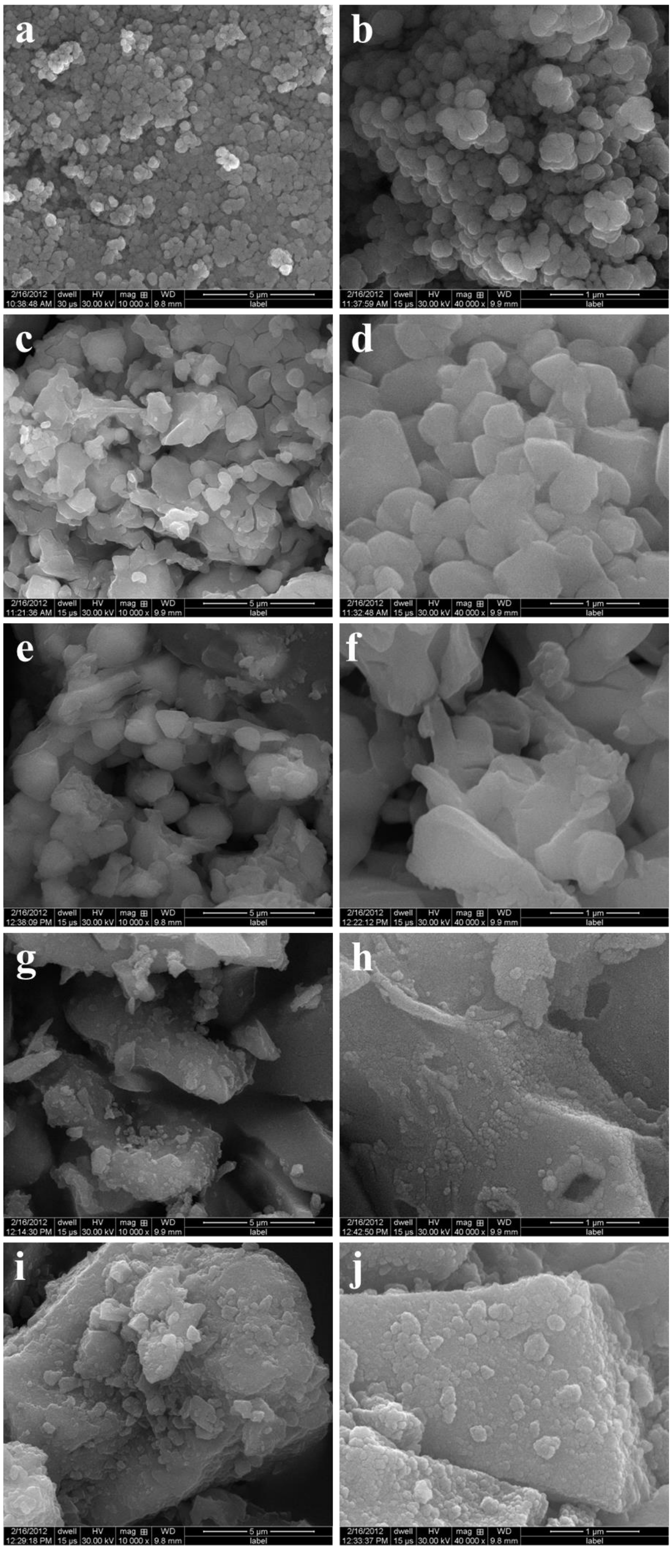

Figure S2. SEM images of (a,b) P25, (c,d) CdS, (e,f) CdS-C_{60}, (g,h) CdS-TiO_2, (i,j) CdS-C_{60}/TiO_2.

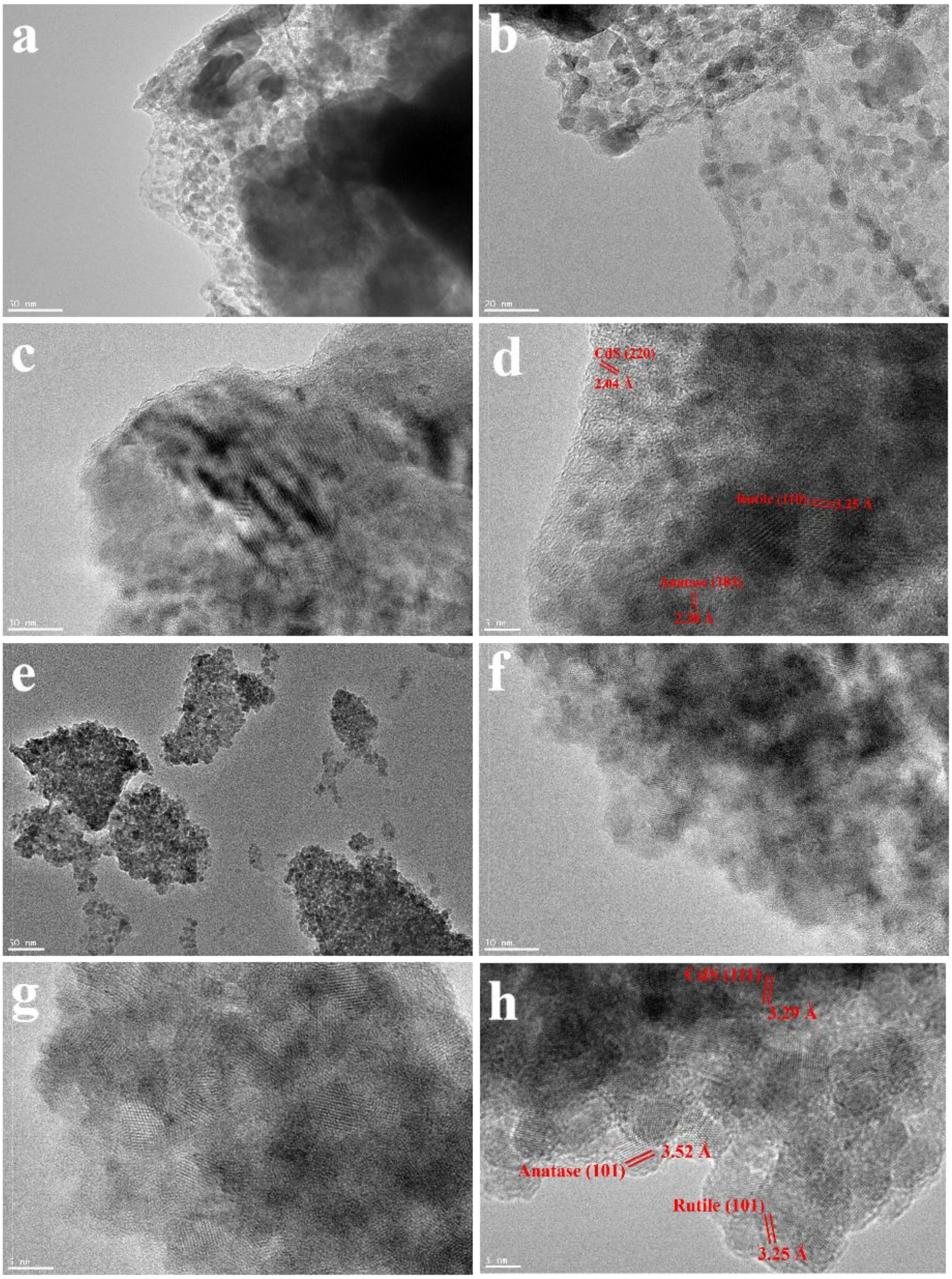

Figure S3. TEM images of the samples of (a,b,c,d) CdS-TiO_2 and (e,f,g,h) CdS-C_{60}/TiO_2.

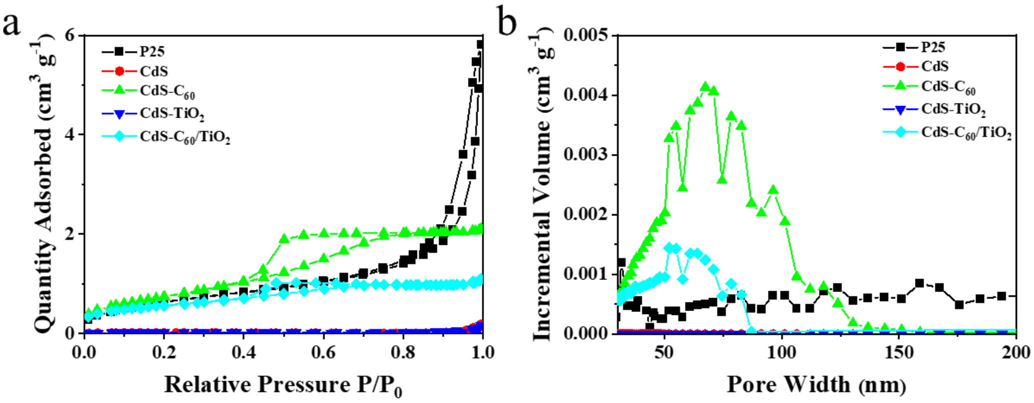

Figure S4. N_2 adsorption and desorption isotherms and pore size distributions of samples.

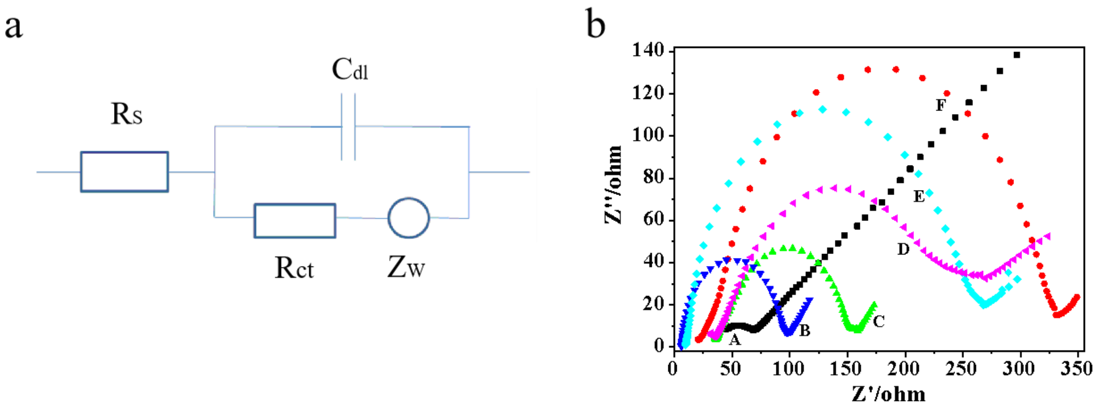

Figure S5. Diagram showing the equivalent circuit for the electrochemical impedance spectroscopy measurement. R_s: electrolyte resistance; R_{ct}: charge transfer resistance; Z_w: warburg impedance; C_{dl}: double-layer capacitance. b) EIS Nyquist plots of A: Au film; B: CdS-C_{60}-TiO_2; C: CdS-C_{60}; D: CdS-TiO_2; E: CdS; F: P25.

Table S1 Kinetics parameters of photocatalytic degradation of MG at different doped TiO_2 nanoparticles

Samples	$Y=\ln(c_0/c)=k_{app}t$	k_{app}(min^{-1})	R	S.D.
P25	Y=-0.0061+6.546E-4*t*	6.546E-4	0.9920	0.0032
CdS	Y=0.0262+0.0156*t*	0.0156	0.9994	0.0200
CdS-C_{60}	Y=-0.0492+0.0202*t*	0.0202	0.9997	0.0202
CdS-TiO_2	Y=-0.0116+0.0026*t*	0.0026	0.9923	0.0119
**CdS-C_{60}/TiO_2**	**Y=-0.0340+0.0303*t***	**0.0303**	**0.9904**	**0.0161**

Table S2 Physical parameters of the adsorption of different photocatalysts

Samples	A_{BET} (m^2/g)	$A_{Langmuir}$ (m^2/g)	$A_{t\text{-}PlOt}$ (m^2/g)	V_{BJH} (cm^3/g)	Pore Size (4*V/A*, nm)
P25	55.0	80.1	67.2	0.204	146.3
CdS	1.2	1.6	0.4	0.006	218.7
CdS-C_{60}	1.6	2.4	1. 6	0.005	142.0
CdS-TiO_2	61.7	87.4	69.1	0.077	47.5
CdS-C_{60}-TiO_2	45.8	63.6	40.7	0.035	33.4

Table S3 The photocatalytic degradation of MG at different catalysts.

Catalysts	kapp (min^{-1})	Time (min)	Degradation (%)	References
$ZnO/ZnFe_2O_4/Pt$	/	120	72	[5]
$CuO-Gd_2Ti_2O_7$	1.98×10^{-2}	90	88.6	[6]
$MgFe_2O_4/Bi_2MoO_6$	1.13×10^{-2}	120	81	[7]
Gd_2O_3/TiO_2	/	60	90.1	[8]
$Co-CNT/TiO_2$	0.59×10^{-2}	300	85.2	[9]
$CdS-C_{60}/TiO_2$	3.003×10^{-2}	120	94.6	This work

References

[1] W.C. Oh, F.J. Zhang, M.L. Chen, Preparation of MWCNT/TiO_2 composites by using MWCNTs and titanium (IV) alkoxide precursors in benzene and their photocatalytic effect and bactericidal activity, *Bull. Korean Chem. Soc.* ***2009***, *30*, 2637-2642.

[2] Y.S. Li, F.L. Jiang, Q. Xiao, R. Li, K. Li, M.F Zhang, A.Q. Zhang, S.F. Sun, Y. Liu, Enhanced photocatalytic activities of TiO2 nanocomposites doped with water-soluble mercapto-capped CdTe quantum dots, *Appl. Catal. B* ***2010***, *101*, 118-129.

[3] AI-Ekabi H, Serpone N, Kinetic studies in heterogeneous photocatalysis. 1. Photocatalytic degradation of chlorinated phenols in aerated aqueous solutions over TiO_2 supported on a glass matrix, *J. Phys. Chem.* ***1988***, *92*, 5726-5731.

[4] H. AI-Ekabi, N. Serpone, X.H. Wang, J.G. Li, H. Kamiyama, Y. Moriyoshi, T. Ishigaki, Wavelength-sensitive photocatalytic degradation of methyl orange in aqueous suspension over Iron(III) doped TiO_2 nanopowders under UV and visible light irradiation, *J. Phys. Chem. B.* ***2006***,*110*, 6804-6809.

[5] G. Korshin, C.W.K. Chow, R. Fabris, M. Drikas, Absorbance spectroscopy-based examination of effects of coagulation on the reactivity of fractions of natural organic matter with varying apparent molecular weights, *Water Res*.***2009***, *43*, 1541-1548.

[6] I. Halomoan, Y. Yulizar, R.M. Surya, D.O. Apriandanu, Facile preparation of CuO-$Gd_2Ti_2O_7$ using acmella uliginosa leaf extract for photocatalytic degradation of malachite green, *Mater. Res. Bull.* 2***022***, *150*, 111726.

[7] J. Jia, X. Du, R.M. Surya, Q.Q. Zhang, E.Z. Liu, J. Fan, Z-scheme $MgFe_2O_4/Bi_2MoO_6$ heterojunction photocatalyst with enhanced visible light photocatalytic activity for malachite green removal, *Appl. Surf. Sci.* ***2019***, *492*, 527-539.

[8] D. Wu, C. Li, D. Zhang, L. Wang, X. Zhang, Z. Shi, Q. Lin, Enhanced photocatalytic activity of Gd^{3+} doped TiO_2 and Gd_2O_3 modified TiO_2 prepared via ball milling method, *J. Rare Earths* ***2019***, *37*, 845-852.

[9] M.F. Zhang, X.F. Liang, Yi Liu, Co-CNT/TiO_2 composites efectively improved the photocatalytic degradation of malachite green, Ionics ***2022,*** 266207600 *https://doi.org/10.1007/s11581-023-05339-7*
